# Sodium butyrate ameliorates intestinal motility and is associated with enteric neuroimmune changes in slow transit constipation mice

**DOI:** 10.3389/fimmu.2026.1852897

**Published:** 2026-09-16

**Authors:** Bingbing Ren, Siqi Fu, Xiangying Wang, Yong Liu, Yuchao Wang, Wei Sun, Chen Xu, Daqing Sun

**Affiliations:** 1 Department of Pediatric Surgery, General Hospital, Tianjin Medical University, Tianjin, China; 2 Department of Orthopedic Oncology Surgery, Shandong Cancer Hospital and Institute Affiliated to Shandong First Medical University, Jinan, Shandong, China; 3 Department of Colorectal Surgery, Tianjin Union Medical Center, Tianjin, China

**Keywords:** enteric nervous system, gut microbiota, macrophages, slow transit constipation, sodium butyrate

## Abstract

**Background:**

The pathogenesis of slow transit constipation (STC) is still not fully elucidated, and there are no related specific drugs currently available for clinical use. Therefore, our study combined 16S ribosomal RNA (rRNA) gene sequencing, untargeted metabolomics, transcriptomic analysis, and a mouse intervention model to characterize the alterations associated with STC and to evaluate the effects of sodium butyrate (NaB).

**Methods:**

16S rRNA gene sequencing, untargeted metabolomics, and RNA sequencing (RNA-seq) were performed in human samples. A loperamide-induced STC mouse model was established to evaluate the effects of NaB. Colonic tissues were assessed using hematoxylin–eosin (H&E) staining, Alcian blue staining, and immunohistochemistry, whereas longitudinal muscle and myenteric plexus preparations were used for immunofluorescence and quantitative reverse transcription PCR (qRT-PCR) analyses.

**Results:**

There were 16 differential bacterial genera between patients with STC and healthy donors. Untargeted metabolomics identified 71 exploratory candidate metabolites meeting variable importance in projection (VIP) > 1 and nominal *p* < 0.05. A total of 2,825 differentially expressed genes (DEGs) were identified. Pathway-level comparison of the independent metabolomic and transcriptomic datasets identified butanoate metabolism as one of the exploratory overlapping pathways. *In vivo*, we found that NaB improved the fecal parameters of STC mice. NaB treatment was accompanied by increased CD206-associated macrophage signals and reduced expression of the pro-inflammatory cytokines in the enteric nervous system (ENS) microenvironment. The number of HuC/D-positive neurons was increased, and the number of HuC/D-positive neurons co-expressing cleaved caspase-3 decreased following NaB treatment. Furthermore, the number of HuC/D-positive neurons co-expressing pSTAT3 decreased following NaB treatment.

**Conclusion:**

NaB improved the constipation-related phenotypes in loperamide-induced STC mice and was accompanied by reduced inflammatory features, decreased myenteric neuronal apoptosis, and reduced neuronal pSTAT3 staining in the ENS. These findings suggest that the beneficial effects of NaB may be associated with the modulation of the enteric neuroimmune microenvironment.

## Introduction

Functional constipation (FC) is a common gastrointestinal (GI) disease mainly manifested by a reduced stool frequency, hard stool, and feelings of incomplete evacuation. These uncomfortable symptoms have serious negative impacts on a patient’s work and life. With changes in dietary patterns and lifestyle, the incidence of FC has increased year by year (1). A recent study confirmed that, compared with individuals without constipation, those with constipation exhibit a 12% increase in all-cause mortality and an 11% and 19% higher risk of developing coronary heart disease and ischemic stroke, respectively (2). In addition, constipation has been associated with mild cognitive impairment in patients with *de novo* Parkinson’s disease (3) and in those with depression (4) and female urinary tract symptoms (5). Slow transit constipation (STC) is a common subtype of FC characterized by a prolonged colonic transit time (CTT) and a reduced defecation frequency. At present, the pathogenesis of STC remains not fully understood, and there are no specific drugs available for clinical use.

Recently, an increasing number of studies have reported that the gut microbiota plays an important role in regulating intestinal motility. In germ-free mice, the enteric nervous system (ENS) exhibited significant structural and functional defects related to gut motility disorders (6, 7). With restoration of the gut microbiota in germ-free mice, not only was the ENS defect modified, but also the gut motility was significantly improved (8). The gut microbiota can upregulate the expression of bone morphogenetic protein 2 (BMP2) by muscularis macrophages (MMs), which contribute to the development of myenteric neurons and improve intestinal motility (9). Gut dysbiosis has been observed in patients with STC (10). In addition, several studies have confirmed abnormalities in the colonic myenteric neurons in patients with STC (11, 12). However, to date, there has been little research on the gut dysbiosis in STC. Therefore, it is necessary to further study the changes in the gut microbiota.

Metabolites are an important type of signaling molecules in the interaction between the gut microbiota and the host. Gut microbiota-derived metabolites, such as *p*-cresol and phenylacetylglutamine, are strongly positively associated with CTT (13). In addition, it has been reported that butyrate, a short-chain fatty acid, could regulate gut motility (14). Furthermore, butyrate contributes to the early development of ENS and neuromuscular transmission (15). Thus, alterations in metabolites may play an important role in the development of STC. To identify changes in the metabolites associated with STC, non-targeted metabolomics analysis was adopted in this study.

Recently, RNA sequencing (RNA-seq) has been widely employed to explore the molecular mechanisms of the pathogenesis of many diseases. In this study, RNA-seq was used to profile the expression of messenger RNAs (mRNAs) in STC colon tissue. It was reported that changes in the gut microbiota can lead to the differential expression of the genes in the mouse intestinal tissue (16). Furthermore, metabolites are also related to the differential expression of genes. Microbial-derived bile acids can modulate the gene expression of the intestinal tissue of patients with inflammatory bowel disease (IBD) (17).

In this study, we combined metabolomic and transcriptomic analyses to identify candidate pathways associated with STC. We further combined 16S ribosomal RNA (rRNA) gene sequencing, untargeted metabolomics, and RNA-seq to characterize alterations in the gut microbiota, metabolites, and gene expression associated with STC. A pathway-level comparison of the independent metabolomic and transcriptomic datasets was subsequently performed to identify candidate pathways. Based on the convergent exploratory findings and the established biological relevance of butyrate to intestinal function, NaB was subsequently evaluated in a loperamide-induced STC mouse model.

## Materials and methods

### Fresh fecal sample collection

The STC patients (STC group) were selected strictly according to the inclusion and exclusion criteria. Healthy donors [control (CON) group] had no recurring GI symptoms. The demographic and clinical characteristics of both STC patients and healthy donors are summarized in **Table 1**. Fresh fecal samples were collected from patients with STC and healthy donors for 16S rRNA sequencing and non-targeted metabolomics analyses. Fresh fecal samples were stored at −80°C until use.

**Table 1:** Characteristics of slow transit constipation (STC) patients and healthy donors.

Characteristic	STC (*n* = 14)	CON (*n* = 12)	*p*
Gender (M/F)	2/12	2/10	0.867
Age (years)	43.75 ± 13.15	47.79 ± 18.06	0.528
Weight (kg)	57.14 ± 4.35	55.46 ± 8.04	0.504
BMI (kg/m^2^)	20.40 ± 2.67	21.79 ± 1.29	0.121
Disease duration (years)	19.08 ± 7.36		
Weekly defecation frequency (times/week)	1.26 ± 0.39	6.92 ± 0.51	<0.001
BSS score (1–7 scale)	1.5 ± 0.52	3.92 ± 0.29	<0.001

*F*, female; *M*, male; *BMI*, body mass index; *BSS*, Bristol Stool Scale.

### Patient criteria

The inclusion criteria were as follows: age ≥18 years; diagnosis of STC based on the Rome IV criteria; CTT >48 h; exclusion of organic large intestinal diseases, megacolon, or colorectal neoplasms via colonoscopy; and disease duration ≥1 year. For healthy donors, inclusion was limited to individuals without constipation, defecation disorders, or organic diseases of the anus and rectum.

The exclusion criteria were: STC due to congenital factors (such as megacolon) or secondary factors (such as drug, endocrine, metabolic, neurological, or psychological disorders); use of probiotics, prebiotics, etc., within the last month; use of antibiotics and/or proton pump inhibitors (PPIs) within the last month; and previous GI surgery and upper GI motor disorders (such as gastroparesis).

### Human colon sample collection

Colon tissues were obtained from STC patients (STC group) undergoing total or subtotal colectomy. Patients with STC were selected according to the inclusion and exclusion criteria. Control colon tissues (CON group) were obtained from patients undergoing colon resection for colon cancer and were collected from macroscopically non-tumor areas located more than 5 cm from the tumor margin. These patients had no history of constipation, defecation disorders, long-term laxative use, or admission for non-acute intestinal obstruction. All colon samples were stored at −80°C for RNA-seq. The characteristics of the patients with STC and the patients with colon cancer are summarized in **Table 2**.

**Table 2:** Clinical characteristics of the patients with slow transit constipation (STC) and the cancer-adjacent controls (CON) used for RNA sequencing.

Patient	Age (years)	Gender	Disease duration (years)	Defecation frequency
STC1	61	Male	20	1/4
STC2	67	Male	23	1/5
STC3	62	Female	15	1/7
STC4	58	Female	13	1/3–5
CON1	71	Male	–	1/1
CON2	65	Female	–	1–2/1
CON3	69	Female	–	1/1

#### Patient criteria

The inclusion criteria were: age ≥18 years, the diagnostic criteria for STC were based on the Rome IV criteria, CTT of >48 h, and disease history ≥1 year. For the CON group, the colon cancer patients had no history of defecation disorder, constipation, long-term laxative use, or admission for non-acute intestinal obstruction.

The exclusion criterion was: STC due to congenital factors (such as megacolon) or secondary factors (such as drug, endocrine, metabolic, neurological, or psychological disorders).

### DNA extraction and 16S rRNA sequencing

Total DNA from fecal samples was extracted using the PowerSoil DNA Isolation Kit (MoBio Laboratories, Carlsbad, CA, USA). After checking the quantity and purity of the obtained DNAcheck, the specific primers with the barcode were used to amplify the 16S rRNA genes by polymerase chain reaction (PCR). Subsequently, the amplified products were analyzed with 1% agarose gel electrophoresis (170 V, 30 min), followed by purification of the PCR products using magnetic beads. Lastly, the library was sequenced on an Illumina MiSeq platform (Illumina, San Diego, CA, USA). Raw reads from sequencing were selected to obtain the high-quality clean tags using Pear (version 0.9.6), Trimmomatic (version 0.36), and Flash (version 1.20) software. Clean tags with sequence similarity greater than 97% were then clustered into the same operational taxonomic unit (OTU). The *α*-diversity indices, including Chao1, Good’s coverage, observed species, Shannon, and Simpson, were calculated using QIIME (v1.8.0). Beta diversity was assessed using principal coordinates analysis (PCoA) based on Bray–Curtis dissimilarity. Differences in the overall microbial community composition between the STC and control groups were evaluated using permutational multivariate analysis of variance (PERMANOVA) with 999 permutations. The taxon-level differences between groups were evaluated using the Wilcoxon rank-sum test, and *p*-values were adjusted for multiple comparisons within each taxonomic level using the Benjamini–Hochberg false discovery rate (FDR) procedure. An FDR-adjusted *p*-value <0.05 was considered statistically significant. Linear discriminant analysis effect size (LEfSe) was performed to determine species with significant differences in abundance between groups.

### Non-targeted metabolomics of fecal samples

Metabolite extraction was performed using a protocol described previously (18). The supernatant from the sample was further separated using an UHPLC system (Vanquish, Thermo Fisher Scientific, Waltham, MA, USA) with a UPLC BEH Amide column (2.1 mm × 100 mm, 1.7 μm). The QE HFX mass spectrometer was used to acquire the mass spectrometry (MS) spectra on information-dependent acquisition mode with the Xcalibur software. Thereafter, the raw data obtained by LC-MS/MS analyses were further screened. The exclusion criteria for metabolite data were as follows: detected in <20% of the experimental samples or in <50% of the quality control (QC) samples, missing values of raw data >50% the minimum value, and relative standard deviation (RSD) >30% in all QC samples. After preprocessing, orthogonal partial least squares discriminant analysis (OPLS-DA) was performed as an exploratory supervised analysis to visualize group discrimination and to derive the variable importance in projection (VIP) scores. Model performance was evaluated using *R*^2^*X*, *R*^2^*Y*, and *Q*^2^, and potential overfitting was assessed using a 200-permutation test. For univariate comparisons, *p*-values were obtained using Student’s *t*-test and adjusted for multiple testing across all 1,287 tested metabolites using the Benjamini–Hochberg procedure. Metabolome-wide statistical significance was defined as an FDR-adjusted *p*-value <0.05. Because no individual metabolite remained significant after FDR correction, metabolites with VIP > 1 and nominal *p* < 0.05 were retained only as exploratory candidate metabolites for hypothesis-generating analyses. Kyoto Encyclopedia of Genes and Genomes (KEGG) pathway enrichment analysis was performed using these exploratory candidate metabolites. Multiple testing correction was also applied at the pathway level, and FDR-adjusted *p*-values are reported.

### RNA sequencing and data analysis

Total RNA was extracted from colonic tissues using RNAiso Plus (TaKaRa Bio, Osaka, Japan). After assessment of the RNA quantity, purity, and integrity, mRNA libraries were prepared using the TruSeq^®^ Stranded mRNA Library Prep Kit and sequenced on the NovaSeq 6000 platform with paired-end 150-bp reads. After removal of adapter-containing, poly-N, and low-quality reads, clean reads were aligned to hg19 using Bowtie2 v2.1.0, and the gene-level read counts were estimated using RSEM v1.2.15. Genes with counts per million (CPM) >1 in at least half of the samples were retained for subsequent differential expression analysis.

The original gene-level raw read count data were reanalyzed using edgeR (version 3.30.0). The library sizes were normalized using the trimmed mean of *M*-values (TMM) method. Differential gene expression between the STC and cancer-adjacent control groups was assessed using the count-based statistical framework implemented in edgeR. Multiple testing correction was performed using the Benjamini–Hochberg procedure, and genes with |log2 fold change| > 1 and FDR < 0.05 were defined as differentially expressed genes (DEGs). Gene Ontology (GO) and KEGG pathway enrichment analyses of the FDR-controlled DEG set were performed using the R package clusterProfiler. Because the colonic transcriptomic and fecal metabolomic datasets were obtained from independent human cohorts, subject-level correlation analysis was not performed. Instead, a pathway-level overlap analysis was conducted to identify the shared KEGG pathways between the transcriptomic and metabolomic datasets.

### Animal experiments

A total of 30 eight-week-old male C57BL/6j mice were purchased from Beijing Hfk Bioscience Co. Ltd. (Beijing, China). All mice were provided with a standard commercial mouse diet and water and a light–dark cycle (12/12 h) at the Tianjin General Surgery Institute. All the animal experimental operations for this study were approved by the Ethics Committee of Tianjin Medical University (no. IRB2026-DW-75).

After a 5-day adaptation period, 30 mice were assigned by the experimenter to three experimental groups (*n* = 10 per group)—the CON group, the loperamide-induced STC group, and the sodium butyrate (NaB) treatment group—before the initiation of the experimental interventions. The mice in the STC and NaB groups were given loperamide (Sigma, St. Louis, MO, USA) by gavage at 10 mg/kg twice per day for 12 days. The CON group was treated with saline as vehicle by gavage. From day 6 to day 12, the NaB group was given 1.1% NaB in the drinking water. Daily water consumption was not prospectively recorded; therefore, the actual NaB exposure in milligrams per kilogram per day could not be calculated. In addition, the health and behavior of mice were observed every day. On day 14, 30 mice were anesthetized via intraperitoneal injection of pentobarbital sodium at a dose of 50 mg/kg. Blood samples were collected by cardiac puncture under anesthesia, and the mice were subsequently sacrificed by cervical dislocation. Death was verified by the absence of respiration and corneal reflex, followed by the collection of colon tissue samples.

### Measurement of the fecal parameters

After day 12 of gavage administration, all mice were fasted for 24 h. On day 13, all mice were gavaged with 0.2 ml of India ink (Yuanye Bio-Technology, Shanghai, China) to observe the time of first black stool defecation, which was used to evaluate GI motility. The stool of an individual mouse was collected during the 6 h after being given India ink. The stool number and wet weight were then recorded. The fecal water content was calculated as previously described (19).

### Gastrointestinal hormone assay

The blood of mice was collected from the heart. The obtained blood was centrifuged at 3,000 rpm for 20 min, and then the serum was collected and stored at −80°C for subsequent analysis. The levels of motilin (MTL) and peptide YY (PYY) in the serum samples were determined using corresponding ELISA kits (96T; Shanghai Enzyme Biotechnology Co., Ltd., Shanghai, China) according to the manufacturer’s instructions.

### Histopathological examination and Alcian blue

Mouse distal colonic tissues were collected and immersed in 4% paraformaldehyde for 48 h. Afterward, the colonic tissues were dehydrated and embedded in paraffin for hematoxylin and eosin (H&E) and Alcian blue (AB) staining. Sections (5 μm) were prepared by microtomes (Leica, Wetzlar, Germany). Distal colonic tissues from all 10 mice in each group were included in the histological analysis. Multiple non-consecutive sections were examined for each mouse. The measurements obtained from multiple sections or microscopic fields were averaged to generate a single value for each mouse, and the individual mouse was considered as the biological unit for statistical analysis. AB was used to assess the thickness of the colonic mucus layer. For AB staining, the percentage of the blue-stained area relative to the total mucosal area was quantified using ImageJ. The measurements obtained from multiple fields were averaged for each mouse before group-level statistical analysis.

### Immunohistochemistry

The distribution of the macrophage-associated markers in colonic tissues was evaluated using immunohistochemistry (IHC). Briefly, sections (5 μm) were dewaxed in xylene and rehydrated in gradient alcohol. Antigen retrieval was then performed by heating in a citrate buffer. After blocking with normal goat serum, the sections were incubated with anti-CD206 (1:500, no. 24595T; Cell Signaling Technology, Inc., Danvers, MA, USA) and anti-F4/80 [1:200; Sangon Biotech (Shanghai) Co., Ltd., Shanghai, China] overnight at 4°C. The next day, the sections were incubated with secondary antibodies for 30 min at 37°C and developed in the DAB horseradish peroxidase color development kit (ZhongShanJinQiao, Beijing, China). For immunohistochemical quantification, multiple high-power fields were evaluated for each mouse. F4/80-positive and CD206-positive cells were counted in each field, and the measurements obtained from multiple fields were averaged to generate a single value for each mouse. The individual mouse was considered as the biological unit for statistical analysis (*n* = 10 mice per group).

### LMMP preparation

The longitudinal muscle and myenteric plexus (LMMP) were prepared as described previously (20). Briefly, the colonic tissues were attached to a silicone plate in ice-cold phosphate-buffered saline (PBS). Beginning at one end of the colonic tissue, the LMMP were separated from the other gut layers using fine forceps. A portion of the obtained LMMP was saved at −80°C for quantitative reverse transcription PCR (qRT-PCR). The remaining part of the LMMP was fixed in 4% paraformaldehyde for immunofluorescence.

### Immunofluorescence

The LMMP were fixed in 4% paraformaldehyde overnight at 4°C and washed three times (5 min each time) with PBS. Subsequently, the LMMP were incubated with 0.5% Triton at room temperature (RT) for 1 h before blocking in PBS containing 5% normal goat serum for 1 h.

For PGP9.5/CD206 double immunofluorescence staining, the LMMP preparations were incubated simultaneously with anti-PGP9.5 (1:500, no. NB600-1160; Novus, Centennial, CO, USA) and anti-CD206 (1:300, no. 24595; Cell Signaling Technology, Inc., Danvers, MA, USA) at 4°C for 36 h. After washing with PBS, the preparations were incubated with goat anti-mouse immunoglobulin G (IgG)–Alexa Fluor 488 (cat. no. abs20013; Absin Bioscience Inc., Shanghai, China) and Cy3-conjugated goat anti-rabbit IgG (cat. no. D110062; Sangon Biotech, Shanghai, China) at RT for 2 h and mounted with a DAPI-containing mounting medium. Fluorescence images were acquired using an OLYMPUS BX51 microscope equipped with a charge-coupled device (CCD) camera and a ×20 objective under identical imaging settings. The myenteric plexus was selected as the region of interest, and the mean fluorescence intensities of PGP9.5 and CD206 were quantified separately using ImageJ software after background subtraction. The measurements from multiple fields were averaged for each mouse, and the individual mouse was considered as the biological unit for statistical analysis.

For the assessment of myenteric neuronal apoptosis, the LMMP preparations were incubated simultaneously with the pan-neuronal marker anti-HuC/D (1:200, no. A-21271; Invitrogen, Carlsbad, CA, USA) and anti-cleaved caspase-3 (1:300, no. 9664T; Cell Signaling Technology, Inc., Danvers, MA, USA) at 4°C for 48 h. After washing with PBS, the preparations were incubated with goat anti-mouse IgG–Alexa Fluor 488 (cat. no. abs20013; Absin Bioscience Inc., Shanghai, China) and Cy3-conjugated goat anti-rabbit IgG (cat. no. D110062; Sangon Biotech, Shanghai, China) at RT for 2 h and mounted with a DAPI-containing mounting medium.

For the assessment of neuronal STAT3 activation, the LMMP preparations were incubated simultaneously with anti-HuC/D (1:200, no. A-21271; Invitrogen, Carlsbad, CA, USA) and anti-pSTAT3 (1:100, no. 9145T; Cell Signaling Technology, Inc., Danvers, MA, USA) at 4°C for 48 h. After washing with PBS, the preparations were incubated with goat anti-mouse IgG–Alexa Fluor 488 (cat. no. abs20013; Absin Bioscience Inc., Shanghai, China) and Cy3-conjugated goat anti-rabbit IgG (cat. no. D110062; Sangon Biotech, Shanghai, China) at RT for 2 h and mounted using a DAPI-containing mounting medium.

Fluorescence images were acquired using an OLYMPUS BX51 microscope equipped with a CCD camera and a ×20 objective. For quantitative analysis, a minimum of 10 myenteric ganglia were selected from each mouse for quantitative analysis. The measurements obtained from individual ganglia were averaged to generate a single value for each mouse. The individual mouse, rather than each myenteric ganglion, was considered as the biological unit for statistical analysis (*n* = 10 mice per group). The numbers of HuC/D-positive neurons, HuC/D-positive neurons co-expressing cleaved caspase-3, and HuC/D-positive neurons co-expressing pSTAT3 were quantified per ganglion.

### Quantitative PCR analysis

Total RNA was extracted from the LMMP using the TRIzol reagent (BS259A; Biosharp Biotechnology, Hefei, China) according to the manufacturer’s protocol. Afterward, cDNA was synthesized using the Hifair^®^ III 1st Strand cDNA Synthesis Kit [11139ES10; Yeasen Biotechnology (Shanghai) Co., Ltd. Shanghai, China]. Subsequently, target gene expression was determined using the Hieff^®^ qPCR SYBR Green Master Mix Kit [1184ES03; Yeasen Biotechnology (Shanghai) Co., Ltd. Shanghai, China]. The genes were amplified under the following conditions: 95°C for 2 min, followed by 40 cycles of 95°C for 10 s and 60°C for 30 s. All primer sequences are shown in **Table 3**. The expression level of the GAPDH mRNA was used as the endogenous reference control. The relative expression of the target mRNA was normalized to the control group by determining the 2^−ΔΔ^*^C^*^T^ values.

**Table 3:** Sequences of the primers used in this study for qRT-PCR.

Gene name	Sequence
*IL-6*	Forward: 5'-CCGGAGAGGAGACTTCACAG-3' Reverse: 5'-TGGGAGTTGCTGTTGAAGTC-3'
*TNF-α*	Forward: 5'-CAGGCGGTGCCTATGTCTC-3' Reverse: 5'-CGATCACGAAGTTCAGTAG-3'
*IL-10*	Forward: 5'-CATGGCCCAGAAATCAAGGA-3' Reverse: 5'-GGAGAAATCGATGACAGCGC-3'
*IL-1β*	Forward: 5'-AAATACCTGTGGCCTTGGGC-3' Reverse: 5'-CTTGGGATCCACACTCTCCAG-3'
*Bax*	Forward: 5'-AGACAGGGGCCTTTTTGCTAC-3' Reverse: 5'-AATTCGCCGGAGACACTCG-3'
*Bcl2*	Forward: 5'-GTGGATGACTGAGTACCT-3' Reverse: 5'-CCAGGAGAAATCAAACAGAG-3'
*Caspase3*	Forward: 5'-TGGTGATGAAGGGGTCATTTATG-3' Reverse: 5'-TTCGGCTTTCCAGTCAGACTC-3'
*Stat3*	Forward: 5'-CAATACCATTGACCTGCCGAT-3' Reverse: 5'-GAGCGACTCAAACTGCCCT-3'
*GAPDH*	Forward: 5'-AGGTCGGTGTGAACGGATTTG-3'
	Reverse: 5'- TGTAGACCATGTAGTTGAGGTCA-3'

### Statistical analysis

Statistical analyses and histograms were processed with GraphPad Prism 9. Data are expressed as the mean ± SD. Significant differences between groups were determined using one-way ANOVA followed by the least significant difference (LSD). A *p* < 0.05 was considered statistically significant. For animal experiments, the individual mouse was considered as the biological and statistical unit. The measurements obtained from multiple tissue sections, microscopic fields, or myenteric ganglia from the same mouse were averaged before statistical analysis.

## Results

### Characteristics of the gut microbiota in STC patients

To explore changes in the gut microbiota in patients with STC, we collected fresh fecal samples from patients with STC and healthy donors for 16S rRNA gene sequencing. A total of 975 OTUs were identified in the two groups, including 866 OTUs in the STC group and 748 OTUs in the CON group. Among these, 639 OTUs were shared between the two groups (**Figure 1A**). We evaluated the *α*-diversity of the gut microbiota using the following four indices: Chao1, Good’s coverage, observed species, and Shannon. There was no significant difference in the *α*-diversity between the two groups (*p* > 0.05) (**Figure 1B**). PCoA showed partial visual differences in the distribution of the gut microbial communities between patients with STC and healthy controls. However, PERMANOVA did not identify a statistically significant difference in the overall community composition (pseudo-*F* = 1.0449, *p* = 0.367; 999 permutations) (**Figure 1C**). We then investigated the differences in the gut microbiota between patients with STC and healthy donors at the phylum and genus levels. At the phylum level, the relative abundance of Fusobacteriota significantly decreased in the STC group (**Figures 1D, E**). At the genus level, 16 bacterial genera remained significantly different between the two groups after FDR correction (**Figures 1F, G**), including *Clostridium_innocuum_group*, *Eisenbergiella*, *Eubacterium_brachy_group*, *Eubacterium_oxidoreducens_group*, *Peptostreptococcus*, *Shuttleworthia*, *Catenibacillus*, *Lachnospiraceae_UCG-010*, *Butyricicoccus*, *Fusobacterium*, *Incertae_Sedis*, *Lachnospiraceae_UCG-004*, *Veillonella*, *Ruminococcus_gauvreauii_group*, *Lachnospiraceae_NK4A136_group*, and *Haemophilus*. To further evaluate butyrate-associated bacteria, we analyzed representative butyrate-associated genera among the differential taxa. The relative abundance rates of *Lachnospiraceae_UCG-010*, *Lachnospiraceae_UCG-004*, and *Lachnospiraceae_NK4A136_group* significantly decreased in patients with STC compared with healthy controls. *Butyricicoccus* also showed a significantly lower relative abundance in patients with STC (**Figure 1H**).

**Figure 1:**
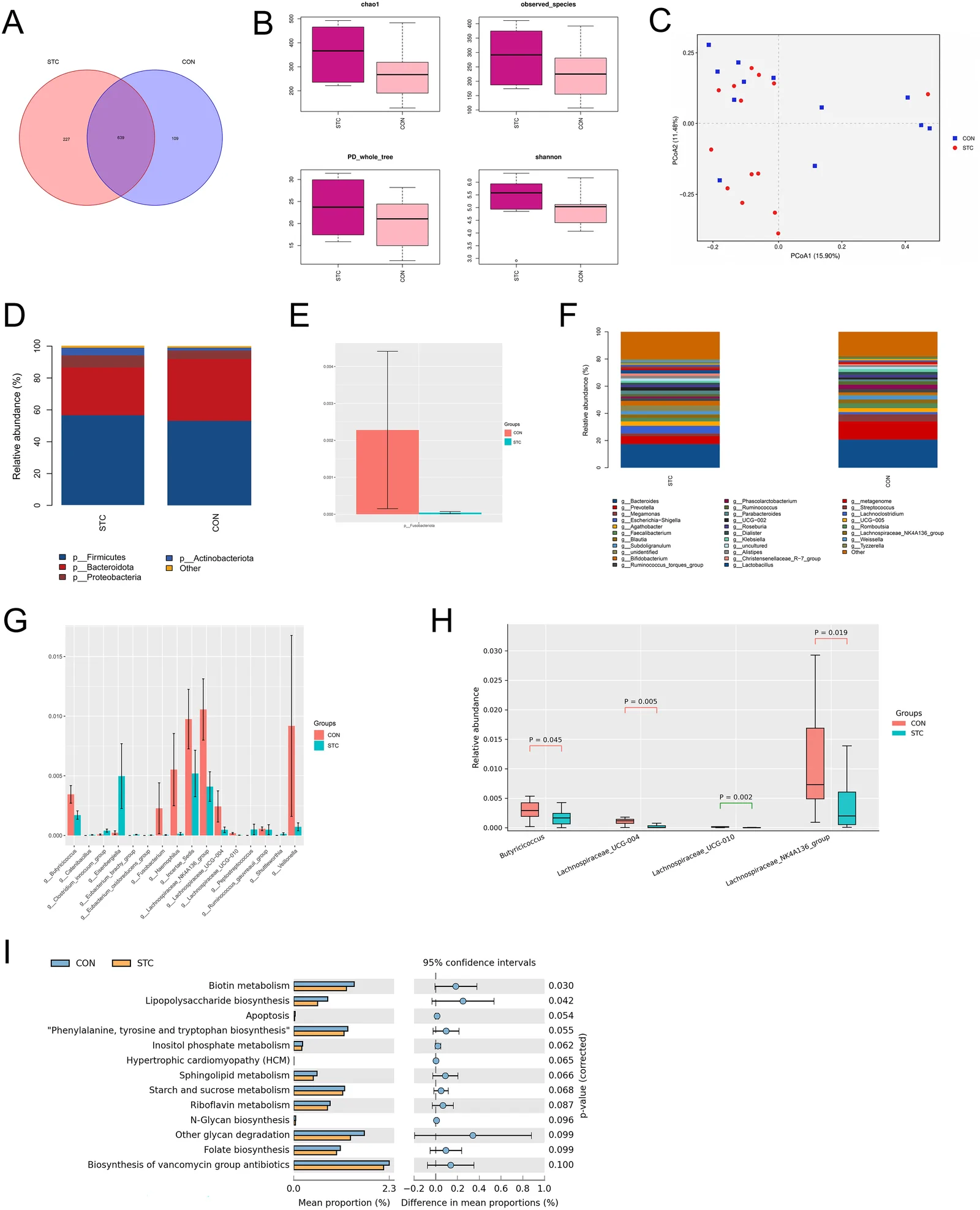
Differences in the gut microbiota between patients with slow transit constipation (STC) and healthy controls. **(A)** Venn diagram showing the common and unique operational taxonomic units (OTUs) in the gut microbiota between STC and healthy controls. **(B)** The indices Chao1, Good’s coverage, observed species, and Shannon reflecting the *α*-diversity within the two groups. **(C)** Principal coordinates analysis (PCoA) of the gut microbial communities based on Bray–Curtis dissimilarity. PCoA1 and PCoA2 explained 15.90% and 11.48% of the variation, respectively. Permutational multivariate analysis of variance (PERMANOVA) with 999 permutations did not identify a significant difference in the overall microbial community composition between the STC and control groups (pseudo-*F* = 1.0449, *p* = 0.367). **(D, E)** Microbiota relative abundance **(D)** and differential flora **(E)** of the gut microbiota at the phylum level between STC patients and healthy controls. **(F, G)** Microbiota relative abundance **(F)** and differential flora **(G)** of the gut microbiota at the genus level between STC patients and healthy controls. **(H)** Relative abundance of representative butyrate-associated genera. **(I)** PICRUSt-predicted functional pathways differing between the STC and control groups.

In addition, we investigated the predicted metabolic pathways of the gut microbiota between the CON and STC groups using PICRUSt. Two predicted metabolic pathways, including biotin metabolism and lipopolysaccharide biosynthesis, differed between the two groups (**Figure 1I**).

### Exploratory characterization of the fecal metabolomic profiles in patients with STC

The influence of the gut microbiota on the host physiology is generally dependent on metabolites. Therefore, we further investigated the fecal metabolomic profiles of patients with STC using untargeted metabolomics. OPLS-DA showed visual separation of the metabolomic profiles between patients with STC and healthy controls (**Figure 2A**). The OPLS-DA model yielded *R*^2^*X* = 0.337, *R*^2^*Y* = 0.992, and *Q*^2^ = 0.183. In the 200-permutation test, the corresponding *p*-values were 0.225 for *R*^2^*Y* and 0.17 for *Q*^2^, indicating that the separation observed in the OPLS-DA model should be interpreted as exploratory rather than as inferential evidence of significant group discrimination. A total of 71 metabolites met the exploratory criteria of VIP > 1 and nominal *p* < 0.05 (**Figure 2B**). However, none of these metabolites remained statistically significant after Benjamini–Hochberg correction for multiple testing (FDR < 0.05). Therefore, these 71 metabolites were considered exploratory candidate metabolites rather than FDR-confirmed differential metabolites. Among these candidates, the metabolites that showed relatively large changes according to the VIP and fold change criteria are presented in **Figure 2C**. We subsequently performed KEGG pathway enrichment analysis based on these exploratory candidate metabolites. Several pathway-level signals were identified (**Figure 2D**). However, after correction for multiple pathway testing, only vitamin digestion and absorption remained statistically significant (FDR < 0.05). Butanoate metabolism was also represented in the pathway analysis, but did not reach statistical significance after multiple testing correction. Using an area under the curve (AUC) threshold of >0.850, five metabolites were identified as candidate discriminatory metabolites: 2-(1-methylpropyl)-4,6-dinitrophenol, argpyrimidine, LysoPE (0:0/18:3), lysyl-leucine, and 2,6-dimethylpyrazine (**Figure 2E**). These metabolites were not necessarily the most significantly altered metabolites by fold change or VIP ranking, but represented metabolites with relatively high discriminatory ability between STC patients and healthy controls in the present cohort.

**Figure 2:**
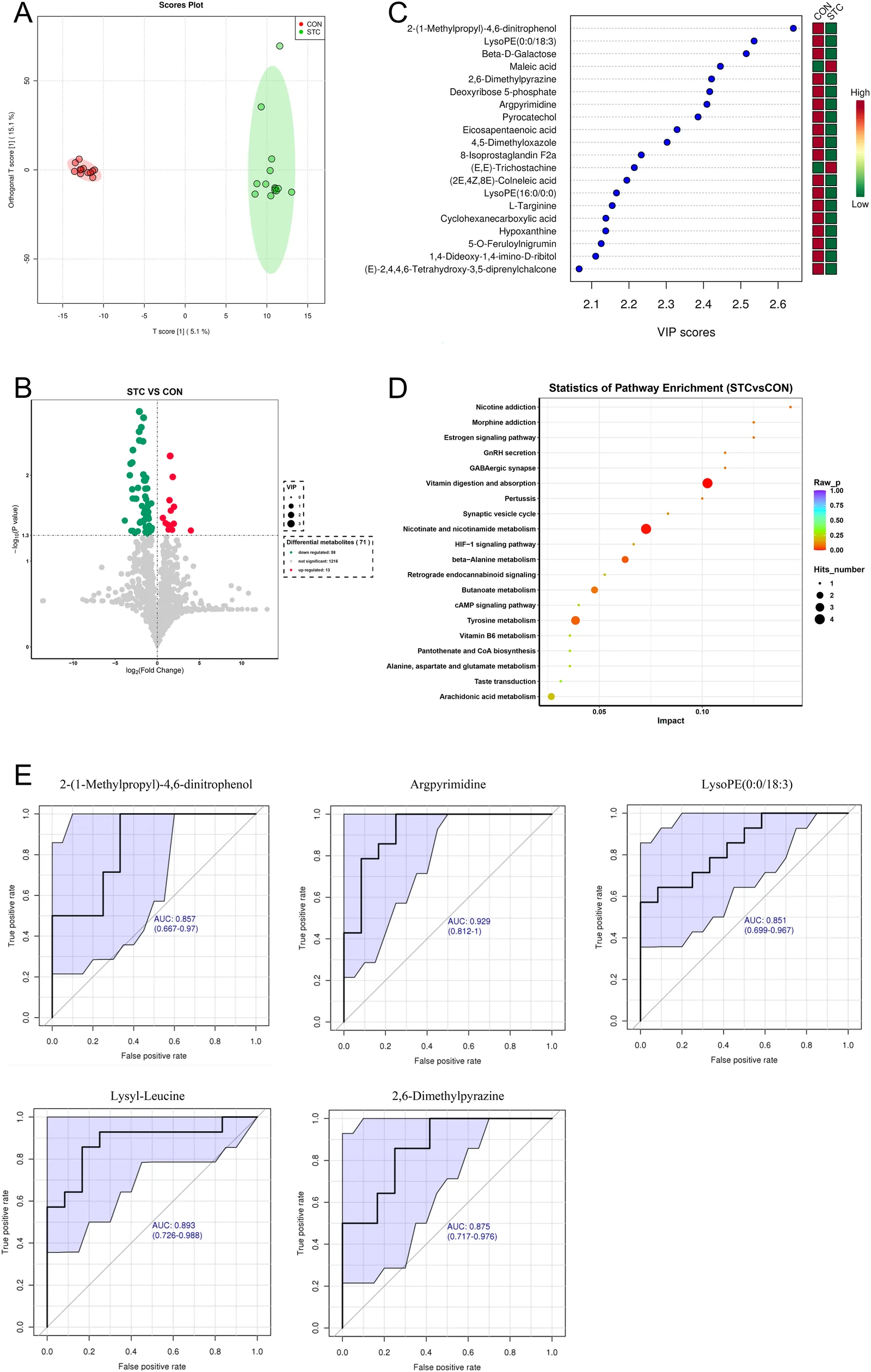
Exploratory characterization of the fecal metabolomic profiles in patients with slow transit constipation (STC) and healthy controls. **(A)** Orthogonal partial least squares discriminant analysis (OPLS-DA) was used as an exploratory supervised analysis to visualize the metabolomic profiles of the two groups. **(B)** Volcano plot showing the exploratory candidate metabolites between STC patients and healthy controls. **(C)** Candidate metabolites showing relatively large changes according to the variable importance in projection (VIP) and fold change criteria. **(D)** Kyoto Encyclopedia of Genes and Genomes (KEGG) pathway enrichment analysis of the exploratory candidate metabolites. **(E)** Exploratory receiver operating characteristic (ROC) curves for five candidate discriminatory metabolites with area under the curve (AUC) values >0.850 in the present discovery cohort.

### Differential gene expression analysis of the colonic tissues in STC patients and cancer-adjacent controls

Although RNA-seq has been widely used in many diseases, it is still rarely used in STC-related studies. To further understand the underlying pathophysiology of STC, RNA-seq was performed to profile mRNA expression. Firstly, it was evident that the gene compositions of the two groups differed from one another based on principal component analysis (PCA) (**Figure 3A**). Using |log2 fold change| > 1 and an FDR-adjusted *p*-value <0.05 as the predefined criteria, a total of 2,825 DEGs were identified. As shown in the volcano plot (**Figure 3B**), the differential genes consisted of 1,460 upregulated and 1,365 downregulated genes. In addition, GO and KEGG analyses were performed. GO enrichment analysis (**Figure 3C**) showed that the DEGs between the two groups are mainly involved in biological processes, such as muscle system process and muscle contraction; molecular functions, such as cell adhesion molecule binding and actin binding; and cellular components, such as adherens junction and actin cytoskeleton. Furthermore, KEGG analysis (**Figure 3D**) indicated that the DEGs are primarily involved in the focal adhesion and protein digestion and absorption signaling pathways. Moreover, butanoate metabolism was represented among pathways containing DEGs. To further identify the pathways shared between the metabolomic and transcriptomic datasets, we performed a pathway-level overlap analysis. Butanoate metabolism and tyrosine metabolism were among the pathways containing relatively large numbers of differential metabolites and DEGs (**Figure 3E**).

**Figure 3:**
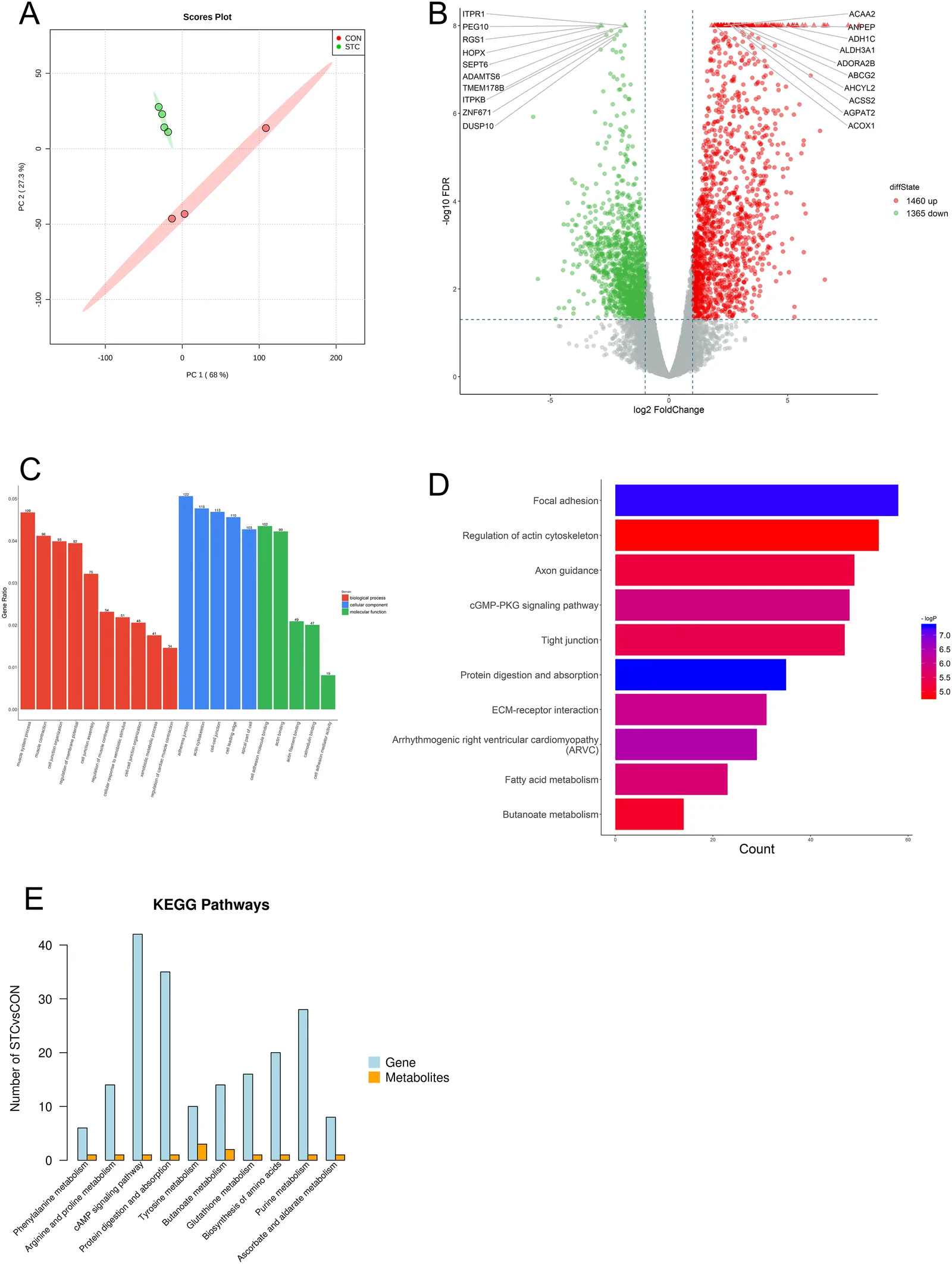
Pathway-level overlap between the independent transcriptomic and exploratory metabolomic datasets. **(A)** Principal component analysis (PCA) of the transcriptomic profiles in the slow transit constipation (STC) and cancer-adjacent control groups. **(B)** Volcano plot showing the differentially expressed genes (DEGs) between the two tissue groups. **(C)** Significantly enriched biological processes, cellular components, molecular functions of the target DEGs identified between the STC and cancer-adjacent control tissues. **(D)** Significantly enriched Kyoto Encyclopedia of Genes and Genomes (KEGG) pathways of the target DEGs identified between the STC and cancer-adjacent control tissues. **(E)** Common differential pathways in metabolomics and transcriptomics.

### Effects of NaB on the fecal parameters, histological structure of the colon, and gastrointestinal hormones

According to the above analysis, we further investigated whether butyrate could improve constipation symptoms and the potential mechanism of constipation improvement. The time to the first black stool in the STC group was significantly longer than that in the CON and NaB groups (**Figure 4A**). After treatment with NaB, the time to the first black stool of the NaB group was significantly shorter than that of the STC group (**Figure 4A**). Furthermore, the number of fecal pellets significantly increased in the NaB group (**Figure 4B**). In addition, the fecal water content and the weight of feces increased toward the control levels in the NaB group compared with the STC group (**Figures 4C, D**). Taken together, these findings indicate that NaB could ameliorate loperamide-induced constipation symptoms in mice. The serum MTL level significantly increased in the NaB group compared with the STC group, whereas the serum PYY level significantly decreased (**Figures 4E, F**). H&E staining showed histological alterations in STC mice, with qualitatively improved mucosal morphology and reduced inflammatory cell infiltration following NaB treatment (**Figure 4G**). AB staining showed reduced mucin-associated staining in STC mice, which increased following NaB treatment (**Figures 4H, I**).

**Figure 4:**
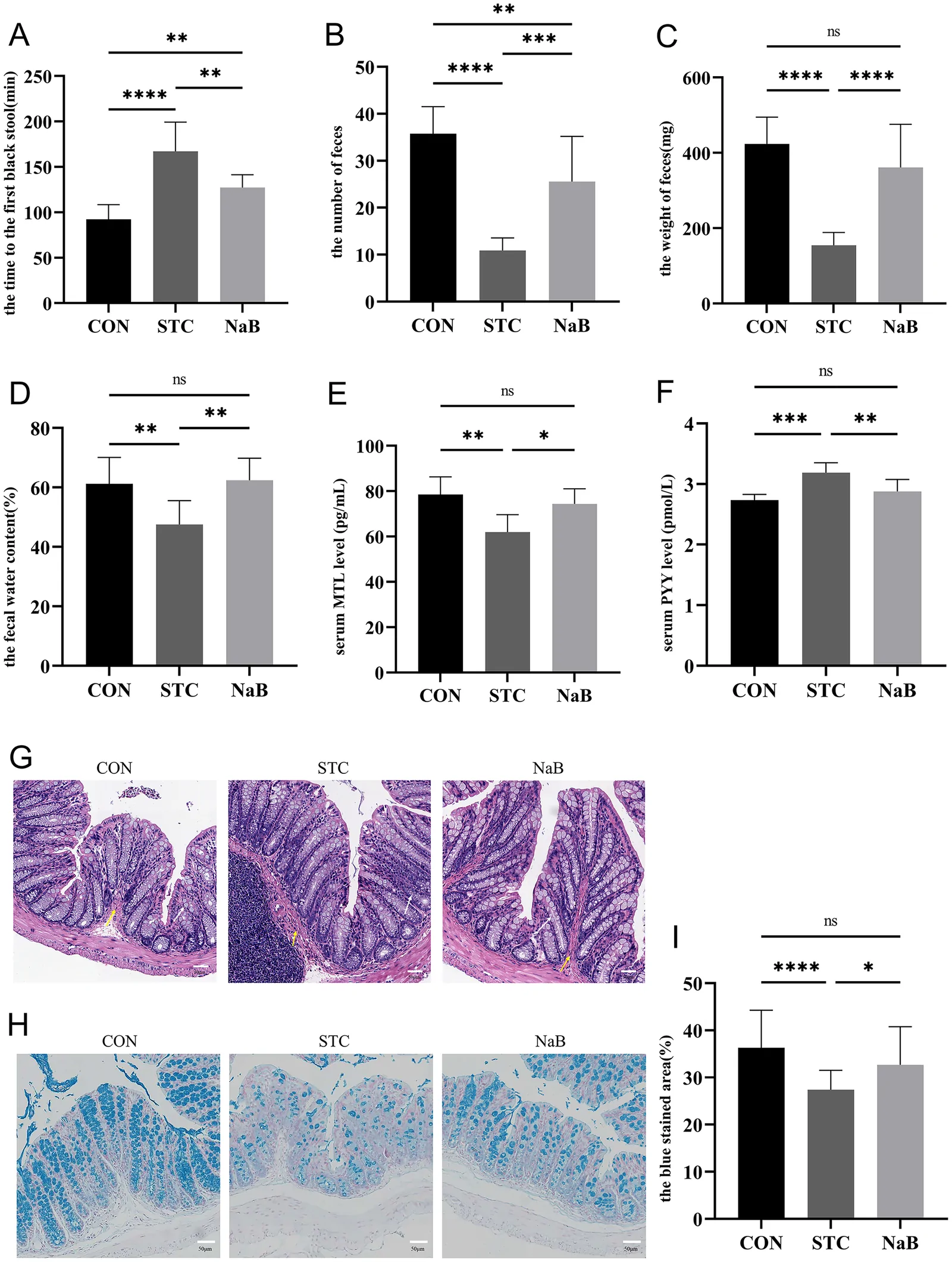
Effects of sodium butyrate (NaB) on the fecal parameters and histological structure of the colon and gastrointestinal hormones. **(A–D)** Effects of NaB on the fecal parameters: time to the first black stool **(A)**, number of feces **(B)**, weight of feces **(C)**, and fecal water content **(D)**. **(E, F)** Levels of the gastrointestinal hormones including motilin (*MTL*) and peptide YY (*PYY*) in the serum of mice. **(G)** Hematoxylin–eosin (H&E) staining was performed to estimate the effect of NaB on the histological profiles in the distal colon. *Yellow arrows* indicate inflammatory cell infiltration, and *white arrows* indicate goblet cells (magnification, ×200). **(H)** Alcian blue staining was performed to investigate the effect of NaB on mucus secretion (magnification, ×200). **(I)** Quantification of the Alcian blue-positive area. *p < 0.05, **p < 0.01, ***p < 0.001, ****p < 0.0001.

### NaB treatment is associated with reduced inflammatory markers and changes in the CD206-associated signals in the ENS microenvironment

The inflammatory milieu of the ENS microenvironment is related to intestinal motility. The macrophage phenotype plays an important role in the ENS microenvironment. Because NaB improved the inflammation in colon tissue, as shown in **Figure 4G**, we examined the macrophage-associated markers in the colonic submucosa and muscular layers. There was no difference in the numbers of F4/80^+^ macrophages in the submucosa and the muscle layer between the CON group and the STC group (**Figures 5A, C**). Compared with the STC group, the F4/80^+^ macrophages in the NaB group significantly decreased in the submucosa and the muscle layer (**Figures 5A, D**). Subsequently, we determined the number of CD206^+^ macrophages in the submucosa and the muscle layer (**Figure 5B**). Compared with the CON group, the number of CD206^+^ macrophages in the submucosa and the muscle layer statistically decreased in the STC group (**Figures 5E, F**). In addition, compared with the STC group, the number of CD206^+^ macrophages in the submucosa and the muscle layer significantly increased in the NaB group (**Figures 5E, F**). Next, we further investigated the change in the inflammatory cytokines such as IL-1β, IL-6, IL-10, and TNF-α in the LMMP. Specifically, the mRNA expression levels of the pro-inflammatory cytokines IL-1β and IL-6 were significantly elevated in the ENS microenvironment of STC mice. Compared with the STC group, the mRNA expression of IL-6 and IL-1β was significantly suppressed following NaB treatment (**Figures 5G, H**). However, there was no significant difference in the mRNA expression of TNF-α among the three groups (**Figure 5I**). Moreover, the mRNA expression level of IL-10 increased in the NaB group compared with the STC group (**Figure 5J**). Double immunofluorescence staining of PGP9.5 and CD206 was then performed in the LMMP preparations to evaluate the neuronal and CD206-associated signals in the ENS microenvironment (**Figure 5K**). Compared with the CON group, the PGP9.5 fluorescence intensity significantly decreased in the STC group; however, it was significantly increased in the NaB group (**Figure 5L**). Compared with the CON group, the intensity of CD206 fluorescence significantly decreased in the STC group. However, the CD206 fluorescence intensity significantly increased following NaB treatment (**Figure 5M**). Taken together, these findings suggest that NaB treatment was associated with the increased CD206-associated macrophage signals and the reduced inflammatory features in the ENS microenvironment.

**Figure 5:**
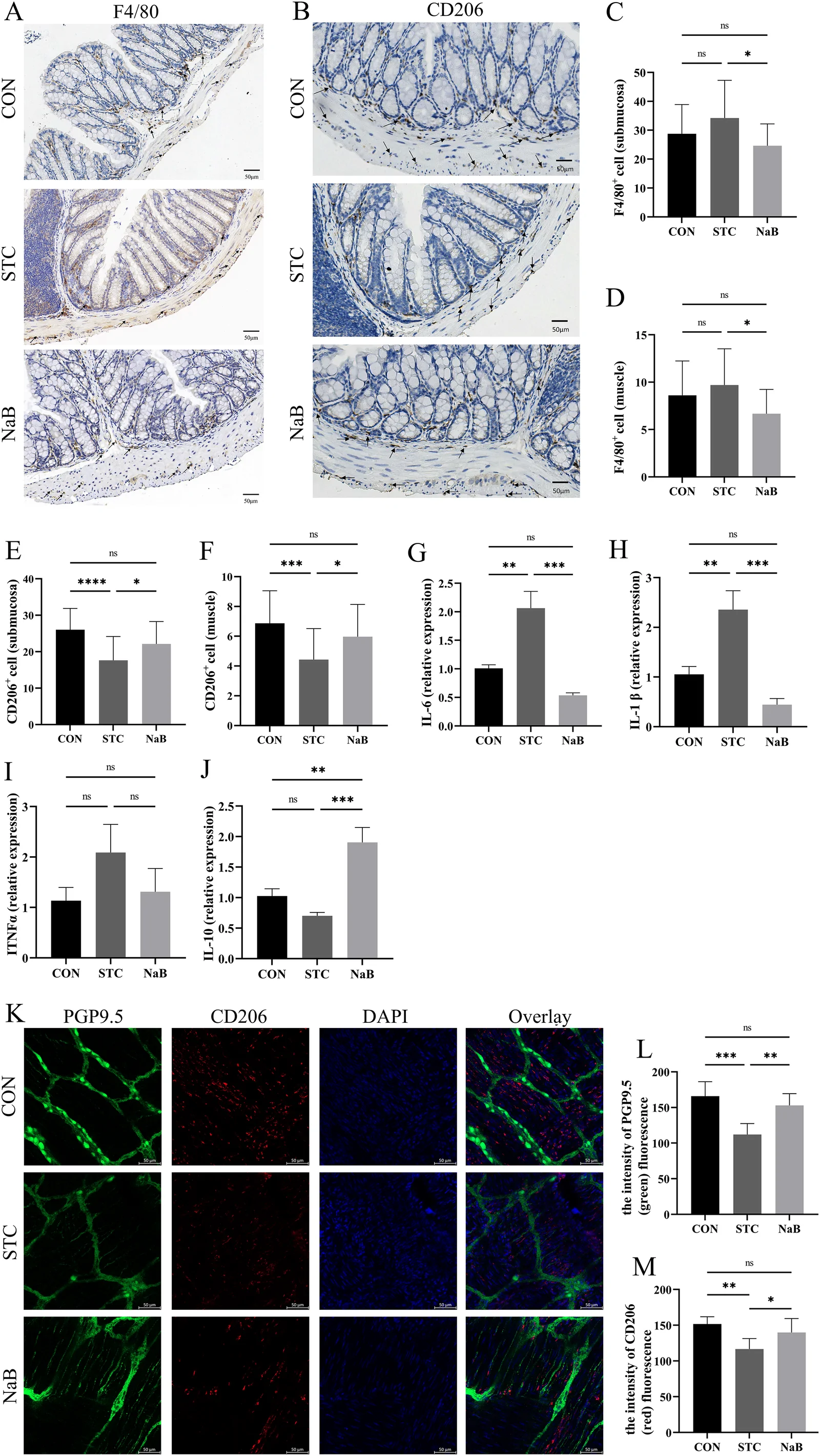
Sodium butyrate (NaB) treatment is associated with reduced inflammatory markers and changes in the CD206-associated signals in the enteric nervous system (ENS) microenvironment. Immunohistochemistry (IHC) was performed to evaluate the macrophage-associated markers in the colonic submucosa and muscular layer (magnification, ×200). **(A, B)** Staining with the F4/80 antibody **(A)** and with CD206 **(B)**. *Thick arrow* indicates positive cells in the submucosa and muscle layer. **(C, D)** Number of F4/80^+^ cells in the submucosa and muscle layer. **(E, F)** Number of CD206^+^ cells in the submucosa and muscle layer. **(G–K)** Changes in the inflammatory cytokines in the longitudinal muscle and myenteric plexus (LMMP): mRNA expression levels of IL-6 **(G)**, IL-1β **(H)**, TNF-α **(I)**, and IL-10 **(J)**. **(K)** Immunofluorescence of LMMP labeled with the pan-neuronal marker PGP9.5 (*green*) and CD206 (*red*). **(L, M)** Fluorescence intensity of PGP9.5 **(L)** and CD206 **(M)**. *p < 0.05, **p < 0.01, ***p < 0.001, ****p < 0.0001.

### NaB treatment is associated with reduced myenteric neuronal apoptosis

Given the changes in the inflammatory markers observed in the ENS microenvironment following NaB treatment, we next assessed the myenteric neuronal number and apoptosis. The number of HuC/D-positive neurons in the STC group statistically decreased (*p* < 0.01) compared with that in the CON group. Following NaB treatment, the number of HuC/D-positive neurons increased compared with the STC group (**Figures 6A, B**). In addition, we found that the number of HuC/D-positive neurons co-expressing cleaved caspase-3 showed a significant increase in the STC group. Compared with the STC group, the number of HuC/D-positive neurons co-expressing cleaved caspase-3 was significantly decreased following NaB treatment (**Figures 6A, C**). Subsequently, we further determined the expression of Bcl2, Bax, and caspase-3 by qRT-PCR. The mRNA expression levels of Bcl2 were upregulated in the NaB group (**Figure 6D**), while those of Bax were downregulated (**Figure 6E**). Consistent with the results of immunofluorescence, we found a statistically significant decrease in the expression of caspase-3 compared with the STC group following NaB treatment (**Figure 6F**). Together, these observations indicate that NaB treatment was associated with the reduced markers of myenteric neuronal apoptosis in STC mice.

**Figure 6:**
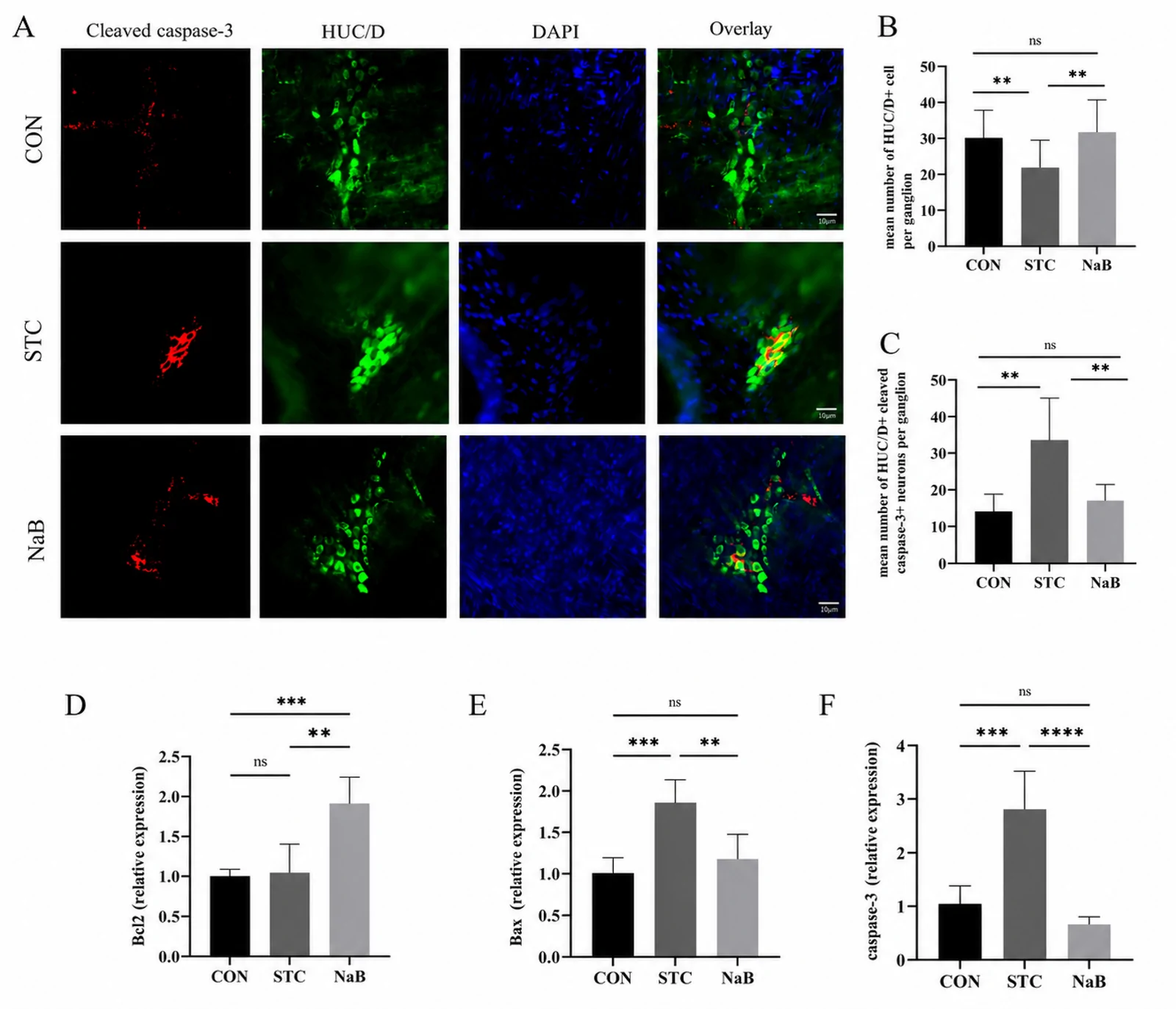
Sodium butyrate (NaB) treatment is associated with reduced markers of myenteric neuronal apoptosis. **(A)** Immunofluorescence of the longitudinal muscle and myenteric plexus (LMMP) labeled with the pan-neuronal marker HUC/D (*green*), cleaved caspase-3 (*red*), and DAPI (*blue*). **(B)** Mean number of HuC/D-positive neurons per ganglion. **(C)** Mean number of HuC/D-positive neurons co-expressing cleaved caspase-3. **(D–F)** Relative mRNA expression levels of Bcl2 **(D)**, Bax **(E)**, and caspase-3 **(F)** in the LMMP. *p < 0.05, **p < 0.01, ***p < 0.001, ****p < 0.0001.

### NaB treatment is associated with reduced neuronal STAT3 activation in STC mice

A previous study demonstrated that inflammation in the ENS microenvironment causes a neuronal loss and delayed intestinal transit by activating the IL-6/STAT3 signaling pathway (21). Given the changes in IL-6 expression and neuronal apoptosis following NaB treatment, we further examined neuronal pSTAT3 staining to determine whether changes in STAT3 activation accompany NaB treatment. Compared with the CON group, we found that the number of HuC/D-positive neurons co-expressing pSTAT3 showed a significant increase in the STC group. The number of HuC/D-positive neurons expressing pSTAT3 decreased significantly in the NaB group (**Figures 7A, B**). These findings suggest that NaB treatment is associated with a reduced activation of neuronal STAT3 signaling in the ENS microenvironment of STC mice.

**Figure 7:**
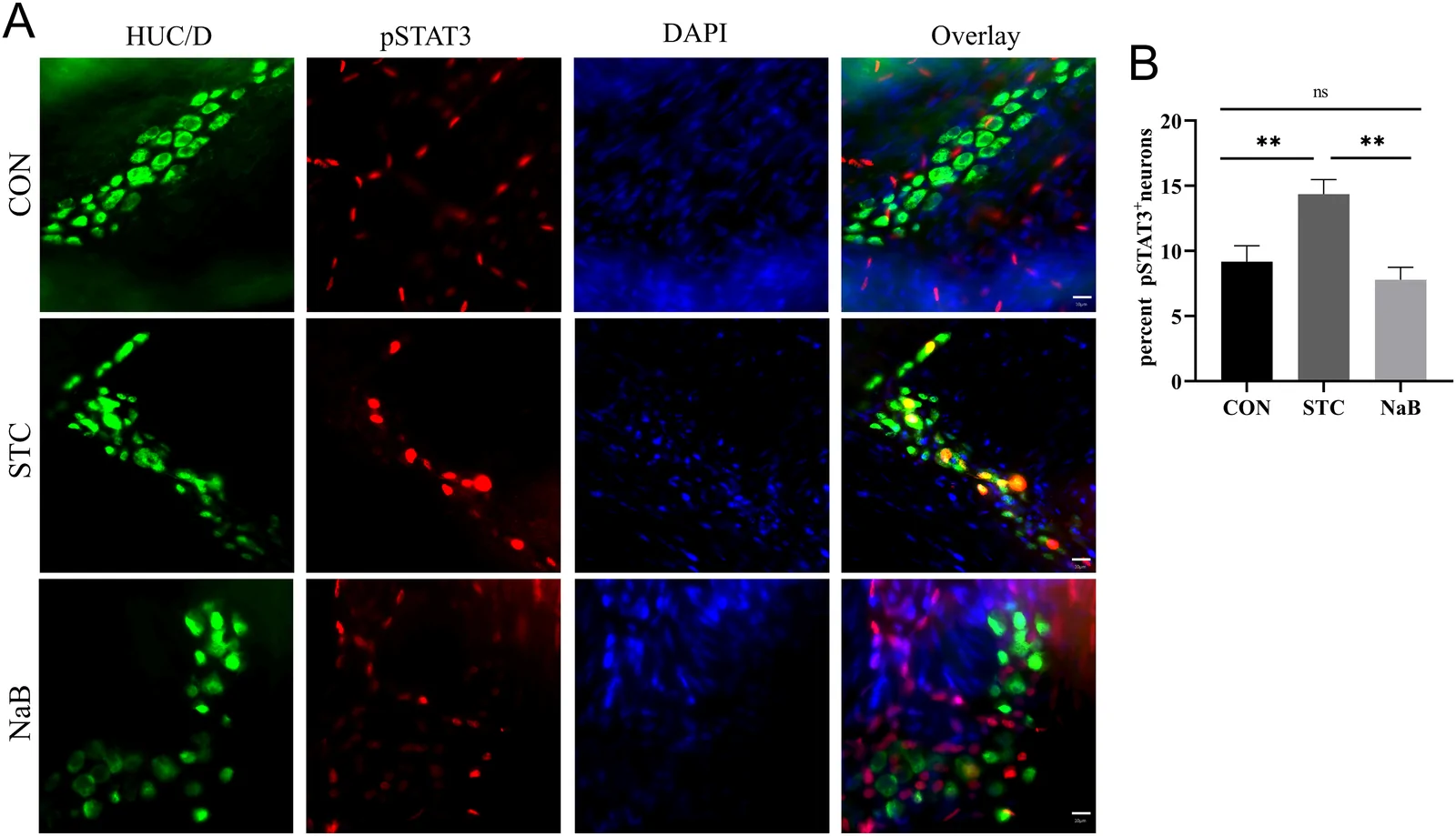
Sodium butyrate (NaB) treatment is associated with reduced neuronal pSTAT3 staining in slow transit constipation (STC) mice. **(A)** Immunofluorescence of the longitudinal muscle and myenteric plexus (LMMP) labeled with the pan-neuronal marker HUC/D (*green*), pSTAT3 (*red*), and DAPI (*blue*). **(B)** The percentage of HuC/D-positive neurons co-expressing pSTAT3 was significantly increased in the STC group. *p < 0.05, **p < 0.01.

## Discussion

In recent years, the gut microbiota has become a research hotspot. However, there is little research on the gut dysbiosis in STC. We collected fresh fecal samples from patients with STC and healthy donors for 16S rRNA sequencing and untargeted metabolomics. Unlike many studies that have found a decrease of *α*-diversity in diseases, here, our study showed that there was no significant difference in the *α*-diversity between patients with STC and healthy donors. However, a number of studies found that the *α*-diversity in patients with STC was increased compared with that of the normal group (10). Based on our results, we believe that the mechanisms underlying gut dysbiosis in patients with STC still need further study.

At the genus level, we found a significant decrease in the relative abundance of *Lachnospiraceae_UCG-010*, *Lachnospiraceae_UCG-004*, and *Lachnospiraceae_NK4A136_group* in patients with STC. As one of the core microflora of the intestinal flora, Lachnospiraceae can produce butyrate and acetate through either the butyryl CoA:acetate CoA transferase pathway or the butyrate kinase pathway (22). Moreover, we found that the relative abundance of *Butyricicoccus* was significantly decreased in patients with STC. The abundance of *Butyricicoccus* was also significantly decreased in constipation-predominant irritable bowel syndrome (23). *Butyricicoccus* is a bacterium that produces butyric acid and has the potential to be a probiotic (24). Therefore, we speculated that the lower relative abundance of these butyrate-associated bacteria may be associated with STC.

To characterize the metabolomic alterations associated with STC, untargeted metabolomics was performed. The metabolomic profiles showed exploratory differences between patients with STC and healthy controls. In addition, we found that the most obvious differential pathway was the vitamin digestion and absorption pathway. At the same time, vitamin B3, vitamin B5, vitamin B6, and vitamin C are involved in the vitamin digestion and absorption pathway. Combined with the results of the non-targeted metabolomics, biotin (vitamin B7) metabolism was shown to be the most prominent differential metabolic pathway of the gut microbiota between the CON and STC groups. The B vitamin group, a water-soluble vitamin, is essential for numerous intermediary metabolic pathways such as neurotransmitter synthesis and neuronal membrane synthesis (25). Therefore, vitamin B deficiency may result in enteric neuron plasticity associated with slow colon transit. Therefore, based on the results of 16S rRNA sequencing and untargeted metabolomics, we believe that the vitamin digestion and absorption pathway may be associated with the occurrence and development of STC. Exploratory pathway analysis showed that butanoate metabolism was represented among the pathway-level findings; however, it did not reach statistical significance after multiple testing correction. Therefore, this finding was interpreted as hypothesis-generating rather than as evidence of a significant downregulation of butanoate metabolism in STC.

Along with changes in the gut microbiota, gene expression profiling of the gut also revealed aberrant expression patterns. It was found that more than 90% of the DEGs in IBD and controls are related to the abundance of microbial taxa only in the healthy state (26). Metabolites are an important type of signaling molecules in the interaction between the gut microbiota and the host. Therefore, we performed a pathway-level comparison between the metabolomic and transcriptomic datasets. At the transcriptomic level, butanoate metabolism was represented among pathways containing DEGs. Although butanoate metabolism was not the top-ranked pathway by enrichment significance alone, it was selected for subsequent functional evaluation due to convergent evidence from the decreased butyrate-producing bacteria, the altered fecal metabolomic pathways, the transcriptomic–metabolomic overlap, and the known biological role of butyrate in intestinal motility and ENS regulation. More recently, a randomized, double-blind, placebo-controlled trial in adults with chronic constipation showed that a multi-strain probiotic intervention improved bowel function and increased fecal butyrate, with the changes in fecal butyrate positively correlated with changes in complete spontaneous bowel movements. Although this population was not specifically characterized as STC, these findings provide supportive human evidence for an association between microbiota-related butyrate metabolism and bowel function (27). Collectively, these findings suggest that butyrate-related metabolism represents one biologically plausible candidate pathway associated with STC and therefore warrants further functional investigation.

Butyric acid is the main energetic substrate of the colonic epithelial cells and presents trophic action on intestinal cells. We found that NaB treatment improved intestinal motility in loperamide-induced STC mice. Butyric acid can ameliorate cardiac function and inhibit sympathetic neural plasticity through the promotion of M2 macrophage polarization in myocardial infarction rats (28). It can also attenuate gut inflammation in mice with dextran sulfate sodium-induced colitis through promoting the expression of the M2 macrophage marker Arg1 (29). In addition, MMs preferentially express the M2 macrophage markers and the tissue protective and wound healing genes (30). Furthermore, it was found that the number of MMs was positively correlated with intestinal motility (31). Therefore, we investigated the number of MMs. We found that the CD206-associated macrophage signals significantly increased after NaB treatment compared with the STC group. Consistent with our previous observations, STC mice showed changes in the macrophage-associated markers (32). In the present study, NaB treatment was associated with increased CD206-positive signals and reduced inflammatory cytokine expression. Alterations in the phenotype of MMs can affect the ENS microenvironment. The LMMP preparations were obtained from the mouse colon, and the mRNA expression of the inflammatory cytokines was assessed using qRT-PCR. We found that the mRNA expression of IL-1β and IL-6 significantly increased in the STC group. Moreover, the mRNA expression of IL-1β and IL-6 significantly decreased following NaB treatment. Although CD206 is commonly used as an M2-associated marker, CD206 staining alone does not definitively establish macrophage identity. Therefore, the PGP9.5/CD206 immunofluorescence findings were interpreted as changes in the CD206-associated signals in relation to the enteric neuronal network rather than as direct evidence of MM localization or polarization.

Next, we further investigated the enteric neuron plasticity by immunofluorescence of the LMMP. It has been found that the apoptotic enteric neurons and neuronal loss increase in patients with STC (11, 33). In line with other studies, we also found that the number of neurons statistically decreased in rats with STC (34). Moreover, our study demonstrated that the number of HuC/D-positive neurons co-expressing cleaved caspase-3 statistically increased in STC mice. After NaB treatment, a significant decrease in the number of apoptotic enteric neurons was observed. In addition, the qRT-PCR findings were consistent with the reduced apoptosis-associated markers following NaB treatment. Taken together, these results suggest that NaB treatment was associated with the reduced apoptosis of myenteric neurons.

Both this study and our previous research found that the mRNA expression of IL-6 significantly increased in the STC group (32). It has been found that STAT3, a downstream mediator of IL-6, is activated in ENS, resulting in neuronal loss and intestinal motility disorder (21). Therefore, we completed immunofluorescence double staining of HUC/D and pSTAT3 to determine the activity of STAT3 in myenteric neurons. Our results showed that neuronal pSTAT3 staining significantly increased in STC mice and then decreased following NaB treatment. However, a significant decrease in the activity of STAT3 in myenteric neurons was observed following NaB treatment. NaB treatment was accompanied by a decreased IL-6 mRNA expression in the LMMP and a reduced pSTAT3 staining in HuC/D-positive myenteric neurons. Together with the reduced IL-6 mRNA expression, these findings suggest that NaB treatment is associated with a reduced IL-6/STAT3-related signaling activity in the ENS. However, the current evidence is insufficient to establish a direct causal role of the IL-6/STAT3 pathway in the beneficial effects of NaB. Future studies using IL-6 stimulation, STAT3 agonists, STAT3 inhibitors, or rescue experiments are required to further clarify this potential mechanism.

Several limitations should be acknowledged. First, the animal experiments lacked a positive drug control, which should be included in future pharmacological validation. Second, the human cohorts were relatively small, particularly the transcriptomic cohort (four STC patients and three cancer-adjacent controls). Moreover, the fecal microbiome/metabolomic and colonic transcriptomic datasets were obtained from independent cohorts, precluding subject-level multi-omics correlation. The use of cancer-adjacent tissues rather than truly healthy colonic tissues as transcriptomic controls also limits the attribution of the observed gene expression differences specifically to STC. Third, no individual metabolite remained significant after FDR correction, and butanoate metabolism did not reach FDR-adjusted significance in the metabolomic pathway analysis. In addition, fecal butyrate was not directly quantified; therefore, the butyrate-related findings should be considered exploratory and hypothesis-generating. Fourth, daily water intake was not prospectively recorded, preventing calculation of the actual NaB exposure in milligrams per kilogram per day. Finally, macrophage depletion or polarization manipulation and direct IL-6/STAT3 intervention or rescue experiments were not performed, limiting causal interpretation of the observed neuroimmune changes.

## Conclusions

In conclusion, this study identified lower relative abundance rates of several butyrate-associated bacterial genera in patients with STC, such as *Lachnospiraceae_UCG-010*, *Lachnospiraceae_UCG-004*, *Lachnospiraceae_NK4A136_group*, and *Butyricicoccus*. Pathway-level comparison of the independent metabolomic and transcriptomic datasets identified butanoate metabolism as one exploratory candidate pathway. In loperamide-induced STC mice, NaB treatment improved the constipation-related phenotypes and intestinal motility. These improvements were accompanied by changes in the CD206-associated macrophage signals, reduced inflammatory cytokine expression, decreased neuronal apoptosis, and reduced neuronal pSTAT3 staining. These findings suggest that the beneficial effects of NaB may be associated with alterations in the ENS microenvironment, although the causal roles of macrophage-associated changes and IL-6/STAT3 signaling require further investigation.

## Data Availability

The analyzed datasets generated during the present study are available from the corresponding author on reasonable request.
